# The Spas of Germany

**Published:** 1837-10-01

**Authors:** 


THE SPAS OF GERMANY. By the Author of "St. Petersburgh." Two Vols. 8vo. with numerous Woodcuts. Colburn, July, 1837.

THE writer of these amusing and useful volumes has wisely cut adrift the long train of titles, honours, and distinctions which were appended to his name on former occasions—shrewdly concluding that one good work outweighs in public estimation a score of titular and honorary decorations—and thereby adopting the sage declaration of the Roman Bard

——————— quæ non fecimus ipsi
Vix ea nostra voco.

The authorship of "ST. PETERSBURGH" is quite sufficient passport to the public to insure a perusal of these volumes, while their fame must rest on their own individual merit. A more popular subject could hardly have been selected than the Spas of Germany. Since the publication of the "BRUNNENS," the road from Coblenz to Frankfort is almost worn to pieces by the carriage-wheels of the English. Our countrymen and women have swallowed more salt water in Germany during the last five or six years than would float the whole Navy of England! How many diseases, disorders, and vapours have been washed out of the British constitution by these drenchings we are unable to calculate; but if only a tithe of those who make the grand tour of the Spas peruse the volumes before us, the sale will be prodigious. Dr. Granville has endeavoured to combine the utile with the dulce—the facetiæ with the scientiæ—the charm of "personal narrative," with the investigation of chemistry, pathology, and therapeutics. We are not prepared to assert that these various qualities are in the best possible proportions. Indeed we suspect that the light, the fugitive, the local, and the temporary incidents are too frequent and prominent in a work which is designed and calculated to issue from the press in successive editions, and to become a stock-book—or we might say, a *starke*-book for travellers in the Spa direction. The author will have many opportunities, we doubt not, to strike out a large portion of *gossip*, in order that more substantial materials may occupy its place.

Be this as it may, it is clear that we must pass over all the facetious, humorous, and piquant anecdotes of these volumes, so as to cull out and concentrate for *our* grave readers the professional information scattered through our author's pages.

### Baden Baden.

Dr. Granville, in order to proceed in the form of "personal narrative," as Humboldt would say, classes the Spas of Germany, not according to their chemical or medicinal qualities, but according to their geographical position in the route which he pursued while visiting them. We attach little importance to the *artificial* classification of *natural* springs. They are generally forced, and seldom useful in practice. Every spring has some peculiarity of temperature or composition which distinguishes it from others of the same class. Our readers are aware that the waters of Baden Baden are upwards of 150° of Fahrenheit. Though cooled down to 98 or 100, there is still much harm done by the almost indiscriminate use or rather abuse of hot baths there as elsewhere, as is acknowledged by Dr. G. himself. A rich merchant was found dead in one of these cauldrons of Medea, and a lady lost the use of her limbs. In our notice of the Baths of Pfeffers, a few numbers back, we adverted to the injury which the human frame is apt to sustain by hot baths incautiously employed.

"There are certain conditions of the body which render these hot baths inadmissible; in such cases, to attempt them, or to persevere in their use, is to rush into danger. Such as have a full, heavy, and distended liver; others who possess very weak powers of digestion, and at the same time suffer from accumulated phlegm in the stomach, and crude or foul secretions in the intestines; a third class, whose strength has been wholly annihilated by a long and dangerous illness; lastly, people naturally inclined to collections of blood in the vessels of the head, and subject to giddiness, palpitations of the heart, or who have had a threatening of apoplexy: all these must abstain from the hot bath at Baden, if they care for their safety." 36.

The above extract, though not worded in the most definite way, exhibits a pretty extensive catalogue of diseases incompatible with hot bathing. But the list might be greatly extended. There are very few chronic diseases where local congestions are not common or even frequent—and what disease, of any standing, is unaccompanied by deranged digestion and "crude and foul secretions?" In fact, the hot bath proves every day, even in this country, a preparatory for the grave! There is scarcely an acute inflammation of any internal structure where the hot-bath is not dangerous—yet there is scarcely one for which it is not daily prescribed by routine practitioners. The following passage points out the physiological effects of the Baden baths on the constitution *in health.*

"A fulness in the head, for which I was obliged to have recourse to the application of cold water to it while in the bath, and a *demangeaison* of the skin during the whole day after, were two, only, of the ill consequences of my trying the *Ursprung,* without any other reason for doing it, than that I wished to ascertain, on my own constitution, the effects of that water. I followed the same method at all the hot-springs, in the course of my long and widely extended peregrinations to the German Spas; and the impression left on the constitution must have been striking, when a noble patient of mine, whom I had occasion to meet at one of the most distant of those Spas, wrote to her friends at home that *the Doctor* had tasted of so many mineral waters, and bathed in so many hot-springs, that he looked as if he had been, first parboiled, and then broiled." 39.

Another effect was an almost irresistible propensity to sleep—an effect produced by the baths of Baden, Wisbaden, Gastein, Toplitz, Carlsbad, Ems, &c. This, in itself, shews the determination which such baths induce to the head. Those physicians who reside at these baths, and who are honest and candid, will be apt to make such disclosures as the above. Baden Baden attracts more visitors who are in search of pleasure than of health. The gambling-tables are well frequented, and we were grieved and disgusted to see some of our fair countrywomen sitting whole evenings at these HELLS, and gambling with as much gout as any black-leg in Regent-street or Pall-mall! A precious school this for the wives and daughters of our aristocracy! In the winter-months families may reside in Baden Baden at a very cheap rate—and several English families do so.

### Wildbad.

This obscure but rising spa is situated near Stutgardt, at the bottom of a valley. We transcribe the following graphic description of a Wildbad bath, in our author's own words.

"After descending a few steps from the dressing-room into the bath-room, I walked over the warm soft sand to the farthest end of the bath, and I laid myself down upon it, near the principal spring, resting my head on a clean wooden pillow. The soothing effect of the water, as it came over me, up to the throat, transparent like the brightest gem or aquamarine, soft, genially warm, and gently murmuring, I shall never forget. Millions of bubbles of gas rose from the sand, and played around me, quivering through the lucid water as they ascended, and bursting at the surface, to be succeeded by others. The sensation produced by these, as many of them, with their tremulous motion, just *effleuraient* the surface of the body, like the much vaunted effect of titillation in animal magnetism, is not to be described. It partakes of tranquillity and exhilaration; of the ecstatic state of a devotee, blended with the repose of an opium eater. The head is calm, the heart is calm, every sense is calm; yet there is neither drowsiness, stupefaction, nor numbness; for every feeling is fresher, and the memory of worldly pleasures keen and sharp. But the operations of the moral as well as physical man are under the spell of some powerfully tranquillising agent. It is the human tempest lulled into all the delicious playings of the ocean's after-waves. From such a position I willingly would never have stirred. To prolong its delicious effects what would I not have given? but the Bad-meister appeared at the top of the steps of the farther door, and warned me to eschew the danger of my situation; for there is danger even in such pleasures as these, if greatly prolonged.

I looked at the watch and the thermometer before I quitted my station. The one told me I had passed a whole hour, in the few minutes I had spent according to my imagination; and the other marked 29½° of Reaumur, or 98¼° of Fahrenheit. But I found the temperature warmer than that, whenever, with my hand, I dug into the bed of sand, as far down as the rock, and disengaged myriads of bubbles of heated air, which imparted to the skin a satiny softness not to be observed in the effects of ordinary warm baths.

These baths are principally used from five o'clock in the morning until seven, and even much later; and again by some people in the evening. The time allowed for remaining in the water is from half an hour to an hour; but it is held to be imprudent to continue the bath to the latter period, as experience has shewn that such sensations as I felt, and have endeavoured to describe, prove ultimately too overpowering to the constitution, if prolonged to excess." 112.

It is natural to ask whence all these heavenly effects of a water which, in every pint, contains only *one grain* of solid matter? It is translucent, tasteless, odourless. Our author thinks that all its efficacy is owing to *temperature*—and we agree with him. But he has recourse to conjecture. "It is not the *thermometrical* temperature—it is the *caloricity* of the water, which is not measured by Fahrenheit—a principle imparted by Nature to the springs in question, from sources which, as yet, have escaped detection." Our author, as if afraid that some critic might here exclaim—

"Nec Deus intersit nisi dignus vindice nodus,"

goes on to a more tangible explanation of the divine effects of the Wildbad waters.

"But there is, in my estimation, a still greater superiority on the side of the Wildbad spring, as a salutary bath, over every other,—no matter how well-managed the latter be; and that is the simple fact, that, whereas in all the other baths the temperature of the water in which the patient is immersed must, and does, progressively diminish, in the course of the hour, or half-an-hour even, during which the operation of bathing lasts—that of the water of the Wildbad bath is uninterruptedly the same, for the water continues in its never-varying natural condition." 116.

Here our author is mistaken. The Baths of Pfeffers, if not many others, are fed by such a profusion, and are so exactly the temperature of the blood, that the waters never sink below nor rise above 98 or 99, for months together. They are constantly running into and out of—that is to say, through, the baths in which the bathers lie.

But Dr. G. has probably attributed too much to the *physique*, and too little to the *morale*, while estimating the delicious sensations produced by the waters of Wildbad. After the fatigues of travel, the novelty of the scene—the expectations that have been raised—the exaggerations of others—and last, not least, our own embellishments, tend not a little to exalt and enhance our actual sensations while bathing and ruminating in the fervid waves of celebrated spas. The delineation of these sensations loses nothing by transcription for the public, however honest may be our intentions, however candid and impartial we may conceive ourselves to be. Our author is well aware of the highly-coloured descriptions of the Brunnens given by a popular author, and how many disappointments were occasioned thereby. Yet no one will suppose that Sir F. Head was blowing "bubbles" for the sake of deception.

But we must rapidly pass over some minor spas included in Dr. Granville's tour, such as Liebenzell, Deinach, &c. The first on the list, indeed, ought not to be entirely overlooked, considering its celebrity in cases of sterility. We believe an illustrious personage of this country visited Liebenzell with the hope of progeny, though that hope was disappointed.

"I have been assured that these baths have been found very useful in scrofulous diseases, and in consumption, accompanied with tubercles; in which latter condition of the lungs, it is said that the exhalation of the surrounding fir forests is also particularly serviceable. In hæmorrhages of all sorts, such as spitting of blood, habitual bleeding from the nose, or from hemorrhoidal vessels, as also where the natural excretion of blood is too profuse, the Liebenzell waters have acquired a well-merited celebrity; and so far, they differ essentially from the Wildbad waters,—as I have shown in the preceding chapter. Used both inwardly, and as baths, they have, of late years, performed some striking cures, in cases of morbid sensibility of the stomach and intestinal canal, and have removed attacks of neuralgia, or *tic*, dependent on that condition of the digestive organs. In female patients, and such of the other sex as possess irritable nerves, or who cannot endure the action of exciting baths, or active medicines, the effects of the *Liebenzell* springs deserve, more attention than they seem to have hitherto received. Dr. Plieninger, the gentleman whom I met at Wildbad, and who is a celebrated accoucheur in Stuttgardt, assured me that he found the *Liebenzell-bad* most strikingly beneficial in female complaints, and that the reputation they had enjoyed for many years, of removing the causes of sterility, was by no means undeserved." 139.

### Bad-Gastein.

Between Munich and Vienna, embosomed in the Noric Alps, and 3000 feet above the level of the Mediterranean, lies the Spa of Gastein—situated in a most romantic locality among rocks, woods, and cataracts. A humorous, and we have no doubt a faithful, sketch is given of Dr. Storch, the physician in ordinary of the spa, and "the Nestor of the spa-doctors in Germany." Dr. G. waited on this venerable Nestor, who was at dinner, being twelve o'clock!

"We caught him, *en demie chemise,* tête-à-tête with his son, eating, at twelve o'clock, his dinner, which lay in the most patriarchal style, partly on his knees, and partly on the only other piece of furniture that appeared to be in the room, besides the chairs. With his mouth full, he welcomed me to Gastein, on hearing my name, and hastened to resume his coat and waistcoat, smiling, at the same time, with an ineffable grin of black-teeth demonstration." 304.

The worthy doctor was found to be more intelligent than was anticipated. But he had one fault. He was infected with Hahnnemania. His conversion to the new light was sudden and almost miraculous. He was prohibited from dispensing medicines and officiating as M.D., at the same time. Another person was, therefore, appointed to dispense. Dr. Storch instantly embraced Hahnnemania, and could then keep a whole pharmacopœia in a snuff-box! The apothecary was ruined! Such, we suspect, is the origin of homœopathy in more places than Gastein.

The water of this spa is of the high temperature of 115°. The vapour rising from its sources is said by Dr. Storch to be wonderfully efficacious in tracheitis and incipient consumption, when inhaled into the lungs. The water itself exactly resembles hot distilled water, having neither taste nor smell. Minute analytical chemistry, however, has detected four-fifths of a grain, nearly, of solid matters in the pint of water. This fraction of a grain is divided amongst twelve different ingredients! Sulphate of soda occupies more than half of this four-fifths of a grain of saline matters. No sensible effect follows the drinking of this water beyond that of ingurgitating any other pure water, and therefore it is only used as a common beverage. The issue of this water is on rather a scanty scale, and hence the cisterns for bathing being filled over night, are seldom emptied in the day. "Few persons, therefore, get a really clean bath"—a comfortable reflection truly! Dr. G. took care to get a first turn at five o'clock in the morning, and consequently had a clean bath. He staid three quarters of an hour.

"The effect produced by the water, on the skin of the hands, during the first ten minutes of being immersed in it, was curious. The bath corrugated and crisped it as if I had held the hands in very hot water for a considerable time; and on passing my hand all over the body, previously to the skin of the fingers becoming crisp—in fact, almost immediately after going into the bath—instead of gliding smoothly and oilily down it, as at Wildbad, it felt ruvid, and the two surfaces seemed to meet with resistance, as if a third body, slightly rough, like the finest sand or powder, lay between. I returned to bed and felt very much soothed and inclined to sleep, but not from any sensation of heaviness in the head or oppression. My breathing was somewhat interfered with, while in the water, sitting upright during the first ten minutes; but this symptom soon vanished. The body did not appear so buoyant in the water as at Wildbad." 322.

The following passage contains a long list of maladies for which the waters of Gastein are famous.

"I have no more doubt of the power which this mineral spring possesses, in the diseases for which it has been recommended, than I have of the effect of bleeding in subduing inflammation. In all complaints which are not connected with increased action of the heart, or with excitement, or (as the German physicians properly term it) with a *morbid elevation of vital activity*, the Gastein baths, judiciously and sufficiently used, will not disappoint the patient. Universal debility, dependent on a derangement of the nervous system, without any apparent inward disease to account for it—depression of spirits and general languor of the constitution, from anxiety of mind—paralysis, in young as well as aged people, consequent on repeated rheumatism, gout, or apoplectic attacks, and such as is produced by irregularities of every sort—affections of the spine—hysteric attacks, and other sufferings owing to sexual disturbance in females—erotic diseases, imperfectly cured—contractions in the muscles of the limbs, or in the joints, and the hip-disease—premature old age—chronic ulcers or eruptions—genuine gout and rheumatism—lastly, derangement of the digestive organs, accompanied by laxity or inactivity of the stomach, or following obstinate diarrhœa and dysentery; these form the catalogue of disorders in which the Gastein water has evinced its marvellous power." 330.

If patients afflicted with any or many of the above catalogue of human ailments (not a few of which are diametrically opposite to each other in their pathology) possess half the faith of our author, we cannot wonder at the miraculous cures that are performed—or, which is the same thing—*said* to be performed! The liquefaction of the blood of St. Januarius is just as *credible*, and authenticated by far more numerous witnesses than the cures by the Gastein!—some of the grave doctors at these Spas believe that a large quantity of the water is absorbed by the bathers, and that its medicinal agencies are thus much greater than if taken into the stomach. This is a mere gratuitous assumption. Dr. Granville *imagines* that the temperature of these Spas being derived from some central volcanic *foyer*, its effects are different from those of temperature artificially created. The worthy doctor does not, however, attach much importance to this hypothesis—and in this we fully agree with him.

### Carlsbad.

This, the king of Spas, is introduced to us by a quotation from the facetious Lord Alvanley's translation of a Latin Ode in praise of the Spa, written some centuries ago. The grand spring here, the Sprudel, astonished our author.

"The sudden view of the violent, lofty, constant, and prodigal up-pourings of hot water out of the bowels of the earth, foaming in the midst of its clouds of vapour, within 45° of the boiling point, on the very margin of a cold, placid, and sluggish stream, the Teple,—rivetted me to the spot for some moments." Vol. 2, p. 6.

The temperature of the water is 167° of Fahrenheit, and contains a large proportion of soda, in different combinations, with an extremely minute portion of iron and a good deal of carbonic acid gas.

"As early as five o'clock in the morning the little nymphs of the *Sprudel*, smart, lively, young lasses, the daughters of poor citizens of Carlsbad, are in attendance to distribute, with critical impartiality, the bounty of their spring. They are all dressed alike, in green dresses in the morning, and in light-coloured robes in the evening: for many invalids attend the Sprudel and the other springs at evening also. Standing at a little distance from the boiling jet, and holding in their hands a stick, four feet long, with a cup-holder fastened at one end of it, these damsels extend the latter towards any one whom they see approaching with his beaker—receive it in their cup-holder—and plunge it within the smoking column of water. From this it is immediately withdrawn quite full, and presented to the same individual again, who, with what relish he may, disposes of its contents. I went through this ceremony with becoming gravity for two or three successive mornings, and quaffed each time half a dozen beakers of the scalding liquid, without making a wry face. Not so with many of those by whom I was surrounded. There were some who sipped, with pigeon sips, the salutary elixir, giving only a gentle shudder as they drank it; and they were ladies. There were others who, more adventurous, swallowed half a beaker-full at once, with only a slight pursing up of the lips, and looked round for approbation; and these were your *esprits forts* and your dandies of the place. My Lord A—— and Sir J. L—— looked grim, when disposing of their dose, and declared it had no taste. Here I detected one, who thought himself unseen, bringing slily out of his large *beniche* a lump of sugar, which he kept in his left hand, ready to pop into his mouth the moment his beaker had quitted it; and he was one of the subjects of Mahmoud, who had not yet exchanged the turban for the ugly and tasselled red cap. There I noticed another, who had just received his beaker-full of the *Sprudel*, from the fair hand of one of the nymphs (who eyed him all the time with a malicious meaning), retire quickly into a corner, his face turned to the wall, where he swallowed in secret the noxious draught; and he was a Polish Jew, by his dress. In this manner I watched the ever-varying modifications of the human countenance, produced under the influence of one and the same agent; and I could not help saying to myself that men are but overgrown babies.

But how humbled and subdued are such *babies* under disease! Look at the air which inward suffering gives to the various individuals here assembled—how meek and unpretending in general! How different would the gait of some of them be, were they in health; and with what difficulty would others bear (were they not made all equal by malady) the temporary association with *every body* and *nobody!* Many of them looked pleased or unoffended, who would have leered superciliously, and annihilated with a scowl, their jostling neighbour, a Jew from Frankfort, or the bearer of a *Plica Polonica,* from Cracow, who was led about by his servant in a complete state of blindness,—so often the concomitant of that disgusting disorder! Instead of this, all seem to go through their hour of trial at the spring together, and harmoniously; envying each other only in proportion to the more or less rapid progress they witness among themselves, towards that restoration to health which they all equally desire. A glance at the vignette of the title-page of this volume will convey, probably, better than my words, a true idea of this *tableau-vivant* of the Sprudel, unique of its kind in Europe." 21.

The Carlsbad waters were chiefly used as Baths till of late years, when their internal use has much superseded their external. From five to eight beakers (six ounces) are generally drunk every morning, as deobstruents or alteratives—for in this quantity the operation on the bowels is slight. The case of an honest Burgher of Prague—Janus Braunes—is mentioned by our author. He has frequented the Spa for eight years past, and drinks from 35 to 40 goblets every morning, and eight or nine in the evening, for six weeks!!

"To prevent mistakes he puts forty copper coins in one of his breeches-pockets when he leaves home, and transfers one of them to the empty pocket of the other side, at each goblet of water he swallows. He is subject to obstinate obstructions of the bowels, experiences at times acute pain in the liver, and his breathing is laborious. On his arrival at Carlsbad he is not able (in consequence of the latter symptom and an excessive corpulency, which he contracts during the winter at home, and which makes him unwieldy) to take any walking exercise; but, before the expiration of the cure, he can ascend all the hills in the neighbourhood, and the circumference of his body diminishes not less than ten or twelve inches." 37.

When we calculate and reflect upon the *quantity* of fluid which, at this and other Spas, is driven through the various vessels and emunctories of the body, it is difficult not to come to the conclusion that it is to *dilution* rather than to medicinal efficacy that the cures are to be chiefly ascribed. Who can doubt that the current which washes away ten or twelve inches of Janus Braune's circumference, must sweep out the Augean stables that are situated within that long line of circumvallation?

"It will be sufficient for my purpose to state generally, that those waters exert their principal sanative action, 1st, on all chronic affections which depend on debility of the digestive organs, accompanied by the accumulation of improper secretions; 2dly, on all obstructions, particularly of the abdomen, which, as Becher, the oracle of Carlsbad, observes, they resolve and disperse; 3dly, on the acrimony of the blood, which they correct, alter, and evacuate, or drive towards the extremities and the surface of the body; 4thly, on calculous and gravelly deposits; 5thly, on many occult and serious disorders, the nature of which is not readily ascertained until after the partial use of the waters.

Looking to this general classification alone, it is impossible, even for the most uninstructed, not to see at once, that nearly two-thirds of the diseases which man, in his civilized state, is heir to, under a lingering, chronic, and often painful form, may find relief in the use of the waters of this Spa; and the reports of all the medical men who have practised at Carlsbad, or have sent patients thither, or have, like myself, visited the place for information, are replete with examples illustrative of the truth of this assertion.

My own experience warrants me in commending the Carlsbad waters in all obstinate cases of induration, tumefaction, tenderness, and sluggish action of the liver; in obstructions of the glands of the mesentery, and in those engorgements of the spleen and distended state of the splenetic vessels, which are much more common (as I proved in another publication) in females, especially of the better classes, than medical men appear to be aware of." 52.

We have now before us a little work on the Carlsbad Waters written by Dr. De Carro an experienced physician, who visits this Spa annually, and whose authority is paramount on the nature and agency of the waters. We make no apology for leaving Dr. Granville for a moment, to introduce the sentiments of the German Physician.

" Soda, as we have seen, being the principal ingredient of our hot springs, their effect can mostly be attributed to that alkali, whatever may be the auxiliary action of their other constituent parts, some of which, discovered later, are in exiguous quantity.

Soda acts with energy on the animal economy, and has a hurtful influence on the arterial system, disposes to hemorrhagies, to scurvy, and disturbs digestion. The Carlsbad water, on the contrary, during the longest cure, animates and vivifies, excites appetite, promotes digestion, and, united to proper regimen, favours the return of health. The difference between the effects of pure soda, and those produced by our waters, cannot be otherwise explained than by the union and combinations of that alkali with their subtler parts, such as the oxide of iron, the carbonic acid gas, and the new ingredients, found by Mr. Berzelius (without speaking of those which may perhaps be hereafter discovered), and particularly by their high temperature, which, volatilizing those constituent parts, so wonderfully united and combined together, conveys them, by the finest ramifications, to the last extremities of our organism, imparts to them a vivifying power, and deprives them of their hurtful qualities. David Becher, whilst drinking our waters, found Carlsbad salt in his own urine, and some drops of his perspiration, gathered upon a watch-glass, examined with a strong microscope, showed, during their evaporation, crystals similar to Carlsbad salt.

It is undoubtedly as difficult to tell by what process alkaline water separates, moves and eliminates morbid matters, as to understand how mercury, introduced by the stomach or the skin, frees the animal economy from inveterate syphilitic affections. Without attempting a methodical solution of this problem, one can conceive how an alkaline substance, reaching, more or less slowly, the last ramifications of our secretory and excretory organs, produces effects not to be expected from coarser medicaments, administered only in small doses, such as antimony, gums, soap, ox-gall, vegetable juices or extracts. Whoever has experienced at Carlsbad a regular crisis, will never more question the power of its waters, the revolution they cause in the whole system, nor the artificial disease excited by such perturbations. The Germans call it, very properly a *Bad-Sturm* (bath-storm).

This hot water occasions neither nausea nor vomiting; it has an exciting action on the stomach, the bowels, kidneys, liver, and other abdominal organs, of which it augments the secretions and excretions. Its action upon the alimentary tube produces a purgative effect; it acts particularly on the blood-vessels, creates often orgasmus and palpitations, and drives the blood to the head. It increases indirectly the activity of the lymphatic system, and it only begins to be tonic, after having brought on secretory and excretory effects. Such are the characters distinguishing Carlsbad from the more chalybeate and gaseous waters, which are more directly tonic.

Abundant purgation is not indispensable to the success of the treatment, and, in many cases, the best results are obtained by copious urine and perspiration; but that all these effects should be simultaneous, is still more desirable. At all events, costiveness should be obviated, and, with that view, we recommend a few additional drachms of Carlsbad salt, Püllna bitter water, glysters and the like.

These various modes of operation have always determined and regulated the use of our waters, and given them the first rank among the remedies commonly called *deobstruent* and *alterative*, in innumerable disorders, proceeding from stagnation and obstructions of the blood-vessels, or of the organs copiously provided with them, from which a variety of abdominal complaints can arise, such as weakness of the stomach, heart-burn, acidities, swelling, eructations, costiveness, which, complicated with derangements of the nervous system, obstruct the liver, the spleen, the mesentery, the epiploon, produce biliary concretions, jaundice in all its degrees, hypochondriasis with its visions, fluent and blind piles, head-ache, vertigo, and all sorts of arthritic, herpetic and urinary complaints.

Hypochondriacal affections appear no where under more various forms than at Carlsbad; and the misanthropic and pusillanimous feelings of those unfortunate beings, passing, without known motives, from hope to despondency, from moroseness to exaltation, deserve the greatest indulgence and sympathy. When we see so many hepatic and splenetic patients whose temper depends entirely on the state of their abdominal functions, we feel disposed to forgive the materialism of the ancients, who placed the seat of so many passions in the liver; we remember unwillingly the *Fervens difficili bile tumet jecur*, the *jecur ulcerosum* of Horace, as synonimous of *jealousy* and *violent love*, and we understand how they could say that men *splene rident, felle irascunt, jecore amant, pulmone, jactantur, corde sapiunt*."*

But to return to Dr. Granville. We were rather surprised at a passage which we met with in page 54 of the second volume, respecting the powers of the Carlsbad waters to resolve *adhesions* of the pleura. The only authority quoted in corroboration of this curious fact (if it be a fact) is the facetious Lord Alvanley, whom our author found one morning sipping the Sprudel, and who assured Dr. G.

"That he had tried it for three seasons, and had, through it, lost a pleuritic adhesion under the *sternum*, or breastbone, the consequence of neglected inflammation in the chest, which had annoyed him for a long time, and had resisted all the curative means adopted in England. The complaint used to make him short-breathed in ascending hills, and gave him a sort of sensation of dragging or *tiraillement* whenever he sneezed; all which symptoms have since disappeared. His Lordship appeared to be quite serious, and not as usual, epigrammatic, on *this* subject." 55.

---

* Essay on the Mineral Waters of Carlsbad. By the Chevalier J. De Carro, Prague 1835.

We suspect that his Lordship's *adhesion* to good eating and drinking was the cause of shortness of breath in ascending hills, and the *tiraillement* when sneezing, rather than the adhesion under the *sternum*. But let that pass. There are other miracles of the Carlsbad waters. Dr. Bigel, of Warsaw, had a large calculus in his bladder, and was operated on, but unsuccessfully, by the lithotritists of Berlin. He repaired to Carslbad, where the waters "smoothed, diminished, and, lastly, expelled," the fragments of stone. This gentleman is a HOMŒOPATH in every thing but Carlsbad waters, in which he is an ALLOPATH.

### MARIENBAD.

We must now shift the scene (some 30 miles from Carlsbad) to the celebrated Kreutzbrunnen, a spa whose medicinal agency has been lauded to the skies, especially by Dr. Heidler, who pronounces it to be "a panacea in all chronic complaints, but particularly those of the digestive organs." Such reputation can only be founded on MONOMANIA, or something worse in the Doctor, or insane credulity in the patients. No sane man can believe in such universal efficacy, whatever may be the agent. Dr. Heidler allows hardly any virtue to the waters of Carlsbad, while De Carro and others have the same opinion of their own panacea, and the comparative inefficacy of Marienbad!

All the waters of this spa are cold, and issue from a peat or marshy soil, or from fissures in the solid granite. They contain free carbonic acid gas, giving them a tartness that disguises, in some degree, their natural saline taste. They feel cold to the stomach, and affect the head like champagne.

"I again drank a glass of the cold water, as I had done the day before. It felt uncomfortable to the stomach for a short time, but walking removed that feeling. Its taste is pleasant and *piquant*. I then mixed it with an equal quantity of the same water heated in stone jugs over a charcoal fire, kept on purpose close at hand. The temperature of the mixture was at first 90°, going down rapidly to 84°, ere I could exhaust the glass. The taste then was exactly like that of veal broth, clear of fat, and with a little salt in it—very pleasant! After the prescribed walks, I again quaffed a second and a third, and, lastly, a fourth goblet of the same mixture, *bien entendu*, on an empty stomach. Dr. Heidler, who was at his post, conversing with and consulted by every one who approached the spring, and to all of whom he had some pretty *rien* to say,—was watching my progress, and my simultaneous examination of the spring and its temperature. He at length came to me, and advised hot mineral water to be added, until the mixture had acquired, by my own thermometer, the temperature of the Sprudel at Carlsbad, so that I could scarcely swallow it. The water had lost, then, the pleasant *goût de bouillon*, and had acquired a stronger, more sapid, more saltish taste, somewhat astringent. Great emission of the gas, or eructations, followed; but the head remained clear, and the digestive organs *still*. The addition of the heated to the cold mineral water instantly destroys its limpidity, and it assumes a gruelly appearance. Ere I quitted the terrace, I had disposed of ten glasses, each containing perhaps six ounces of water. After such a performance, I proceeded to breakfast, with what appetite I could; and *mum* as to the result. A solvent effect of the water had been required for four days by the patient, who is not generally fond of, nor constrained to use, his own physic while travelling."

We have already alluded to the unbounded reputation of this water in disorders of the digestive organs. It is diurectic and purgative in the quantity of from four to eight beakers, or six-ounce glasses. Dr. G. thinks it more efficacious when heated :—

"On the action of the heart and the pulse this water exerts considerable influence. At first it seems to disturb both; but as soon as its effect on the intestines is established, that disturbance ceases, and a well-being ensues, which is not readily obtained from the daily use of ordinary drugs. This property of the *Kreutzbrunnen* renders it susceptible of application in cases where either the constitution or the age of the patient admits of no remedy productive of irritation; and, therefore, persons subject to apoplexy even, or such as are threatened with consumption of the lungs, may take the *Kreutzbrunnen* mixed either with hot mineral water or milk, for the removal of those disorders." 129.

Our author in the above, and in many other places, uses rather loose language for the sake of the general reader. Thus a person *subject* to apoplexy, means one who has often had the disease. We would not say that a man was *subject* to winter-cough, for example, unless he had had the complaint during several Winters—and so of hæmoptysis, or any other disease. Surely Dr. Granville would never recommend the Marienbad waters, which act like champagne on the brain, to a person who had had apoplectic attacks. The word ought to have been *predisposed;* but even in case of predisposition, we should be very sorry to prescribe these waters, or any of the kind. Dr. G. has much confidence in these waters in amenorrhœa; at least we conjecture that this is the affection he alludes to, though the language is so very refined that it is tinged with the obscure. "On several of the affections *peculiar* to the female constitution, the influence of the Kreutzbrunnen is well marked and indisputable. A noble lady, the Marchioness of—, who was still at Marienbad during my visit, and Mrs. C—, may testify to this fact." Quere, What fact? The Marchioness and Mrs. C. may have laboured under menorrhagia, amenorrhœa, sterility, ovarian dropsy, scirrus uteri, or many other *affections* peculiar to females; but we are left entirely in the dark as to which of these affections the fair witnesses could testify to. Whether the following passage will throw any light on the subject, we will not say:—

"I consider the water of three of the springs at Marienbad to be sufficient for the removal of most of the disorders of this class;—that of the first by acting as an expurgator and solvent; of the second by giving tone to vessels hitherto lax or paralyzed; and of the third by forcing, as it were, nature into a proper performance of its functions, after the removal of disease. But each case must be well studied, and care taken that the patient understands the manner, as well as the object, of the treatment."

Dr. G. does not consider the waters of Marienbad as adapted for gouty or rheumatic patients. The Kreutzbrunnen is, in his opinion, tonic and excitant, while it is a powerful solvent. They are ferruginous and saline. As to Dr. Heidler, the medical oracle of the place, Dr. G. considers him, and justly, as an enthusiast—"too blind a worshipper" of the spa to be implicitly relied upon, unless "taken *cum grano salis*" (*Glauberi*, of course), when he may prove a useful guide. We should prefer taking the salts by themselves to taking them with Heidler into the bargain. These waters are often used as baths, generally tepid, when they excite the surface even to redness, when not more than 90°. The most extraordinary feature of this spa, however, is the gas-bath, a remedy first employed, with happy effects, on himself by Dr. Struve, of Dresden.

"The success of this first attempt having been made known, numerous were the applications of patients for the use of the gas; but as the exposure of the limbs to the column of gas over the *Marienbrunnen* was found inconvenient, the present establishment was erected, in which more than one patient may employ the gas-bath, either generally or partially. For the former, the patient enters a wooden pan or tub, covered with a wooden lid, and sits on a stool perforated with holes, when the gas is admitted through pipes as it issues from the earth. The head is outside, and the aperture of the lid around it is lined with cloth, in order to prevent the injurious effects which the escape of the gas might produce on the head and lungs.

I tried the effect of the gas against my hands and face, as it issued from the earth through a pipe, with as much force as if some one had been blowing on those parts with a pair of bellows. After the first sensation of cold, that of genial warmth followed, and the heat of the part continued for some time after. I received some of the gas into my mouth, and instantly felt the powerful effect of the compound gaseous mixture. My respiration became affected, and three or four darts of acute pain in the head followed." 137.

Marienbad is rising in reputation, and consequently in riches. The *sejour* of royalty, and a sprinkling of German and Russian nobility, give a *ton* to the spa, which, however, is not a place of pleasure, but of health.

### EGRA, OR EGER.

This is but a morning's drive from Marienbad. The spring is of the temperature of 53°, Summer and Winter, pervaded every where by gas-bubbles, and limpid as crystal.

"The predominant saline ingredient is the sulphate of soda, or Glauber salt, of which there are twenty-five grains in sixteen ounces of the water, with nearly eight and a half grains of muriate of soda, or common salt, and the same quantity of bicarbonate of soda. The proportion of iron present is a little more than the third of that contained in the *Kreutzbrunnen*, and just one-sixth part of the quantity held in solution by the *Ferdinandsbrunnen* of Marienbad. The whole of the solid contents in the sixteen ounces of water weighs forty-four grains and a fraction." 151.

Dr. Koestler, "instructed me—and that too in the course of a few hours—on every subject of the medical practice at the spa." This was quick work certainly. We know Dr. G. to be an apt scholar, and keen as a razor; but the above instruction amounts almost to intuition. "Diseases affecting the organs of digestion, and those of secretion and excretion, the lymphatic vessels and glands, as well as the mucous membranes, are principally benefited by either water." These two springs are the Salzequelle and the Franzensquelle, between which the greater number of visitors vibrate early in the morning. The Franzensquelle is too stimulating where there is any inflammatory affection or nervous irritability. The Salzequelle is, in such cases, preferable.

"The MUD-BATHS of Franzensbad are, in my opinion, next to the gas-baths, an object of the greatest importance; and they are peculiar to that Spa. This is owing to the particular energy and power possessed by the materials which nature has here presented to the physician, and which in other places—as at

Carlsbad and Marienbad, where the same contrivances have been attempted—are of so inferior a quality, that they will never deserve any serious consideration, whatever efforts my friends, De Carro and Heidler, and their colleagues, may make for that purpose. 'In a few years,' observed, to me, Dr. Koestler, as we were coming out of the great bathing-establishment, 'I predict that Franzensbad will be the most celebrated Spa in Germany, merely on account of its mud-baths. They are specific and infallible in all cases of excessive debility and prostration, particularly in paralysis, of which gout has been the cause. I am willing (added he with great emphasis) and ready to take upon myself all the expenses of any paralytic patient of that class who shall arrive here, and fail to be cured, after a proper use of the mud-baths. When the Toeplitz doctors find that they cannot cure, with their hot springs, patients affected by paralysis, they are glad to hand them over to our mud-baths.'" 160.

Dr. G. afterwards saw a young lady who had arrived at Franzensbad, "with her legs and feet completely paralyzed, and who, after six immersions in the mud-bath, could walk without assistance." Dr. G. avers, that a mud-bath "made of such stuff as he saw at Franzensbad, is an agent of infinite, indeed of *almost* dangerous power." The stuff is quarried in a neighbouring field, by means of spades, where he saw a stratum of nearly 20 feet deep, immediately below the turf, lying on a bed of sand. The mass is moist, of a beautiful jet black colour, curiously stratified with thin plates of iron pyrites. This stratum is plentiful for miles around Franzensbad, rendering the whole surface tremulous, like a quagmire. It reddens litmus paper, and it tastes acid in the mouth. The smell is that of sulphureous acid gas. This bog-earth is brought into the court-yard of the bathing establishment, and thrown into two large wooden vats, where it is diluted with hot water from the Louisenbrunn, till it is of proper consistence and temperature for bathing. The temperature is about 80°, and the consistency of the bath is that of a semi-fluid bread poultice.

"It exhales a smell not unlike that of pyroligneous acid, which penetrates the skin to such a degree that many hours afterwards the surface of the body will taste of it. On coming out of the bath, and after having washed the mud off with warm mineral water, the skin feels soft and looks almost like satin. Dr. Clarus of Leipsig, who has studied these baths, observes of them, 'that they increase the action of the skin, are solvent as well as emollient, and stimulate the nervous system;' which effects he attributes to four principal elements found in the composition of the mud: 'The fatty and peaty matter;—caloric;—the presence of a highly volatile substance;—and lastly, the metallic and saline ingredients.' When the *boue*, after having been used, is thrown away, it will, in a few moments, exhibit on its surface strata of half an inch in thickness of sulphate of iron and Glauber salt, and present also a large quantity of free sulphuric acid. Unquestionably the *Franzensbad* doctors have, in this material, a most powerful agent at their command, for combating disease with great success." 164.

But the gas-baths here are, in our author's opinion, more efficient than the mud-baths. A tube is plunged into the ground, and gas instantly issues through it, which, being conveyed by a horizontal pipe into a bathing-tub, falls in showers, as it were, upon the ailing part of the body exposed to it under cover. Scrofulous ulcers are said to be quickly healed by this application.

PULLNA, SEIDLITZ, &c.

The waters of Pullna acquire their medicinal powers while standing in the wells. When the latter are first dug, the water is pure and tasteless; after a sojourn of a few days, it is found to contain nearly 90 grains of solid ingredients in the pint; viz. carbonate and muriate of magnesia, sulphate of potash, Epsom and Glauber's salts. No trace of iron is found in the waters of Pullna. This water has a bitter taste, and aperient qualities. The following extract is in Dr. Granville's peculiar style of sentiment.

"About ten years ago, on passing through the neighbourhood of Pullna, heated from a journey of two thousand miles, performed without stoppage by night or by day, and from that habitual state of the body for which I had, like many more of the inhabitants of London, taken bushels of aperient pills, in the preceding years,—I was induced, on principle, to try the effect of the *Bitterwasser*. The result of that trial was, that I recovered my health—conquered my habitual difficulty of digestion—abandoned all pills—have never taken any since —and find it necessary only to drink about two ounces of the Pullna water to keep all straight. But as the real Pullna is not to be had easily in England, and is not very good when one gets it, I had recourse to the artificial one prepared at Brighton, without detecting, either in taste or in its effects, at first, or at any time since, the slightest difference between the natural and the artificial water. The latter I have therefore been in the habit of recommending, with the greatest success, in many cases analogous to my own; and I will be bold to say that, were its virtues better known, and its use more general, we should not hear of half the complaints against those endless pills and draughts for cleansing the bowels, with which most of the medical visits conclude in England." 187.

If, as is threatened, a fountain of Hygeia should be opened in the Regent's-park, the Company in Bridge-street may shut up shop! Dr. Struve will not be able to supply the metropolis with Pullna water, even if the New River be turned in under the portals of the Colosseum.

The Seidschutz is near Pullna, and is "an effectual purgative," and "may be substituted with effect for many *messes* and *compounds* from the apothecaries' shop, in cases of obstruction and slight attacks of hepatic disorders." It contains the slightest trace of iron, and the solid contents of a pint amount to 130 grains.

As for the SEIDLITZ waters, we need not dwell upon them, as their imitation (Seidlitz powders) are familiar to all. The *real* Seidlitz, however, is bitter to the taste, whereas the imitation is acid. The former contains Epsom salts, the fictitious none. Nevertheless, the said Seidlitz powders are very useful and pleasant aperients.

TOEPLITZ.

This is essentially a bathing-place. The waters are at a temperature of 122°. They are taken internally also, for suppressed gout, chronic rheumatism, &c. They contain much carbonate of soda—and also of iron, which is deposited as the waters cool down.

"With proper management I should not despair of recovering from all his ailments, the most pitiable object of gouty tyranny."

This is a bold assertion, and we doubt not that if Dr. G. can realize only half his promise, he will soon be the richest physician in this metropolis.

"The scene that presented itself on entering the bath of the men, as well as the separate one of the women (both of which were *quite full*), under the guidance of Dr. Bischof,—who insisted on my doing so, and who quieted the incipient alarm of the ladies, by exclaiming that I was *only* a doctor,—beggars description. The heat of these baths was 113°, and the steam almost prevented a distinct view of the objects in it. Still I could perceive that both the men and the women, who were bathing, had been cupped on, or had had leeches applied to their backs, their shoulders, or their chests, and that blood was streming from the wounds, fresh and free, into the water. These poor creatures, who have all an equal right to use these baths gratuitously, cannot enter the water, on account of its great heat, without first losing blood, or they would expose themselves to serious accidents. Some were lying down in the water; others standing up; a few were playing and gamboling about; while many were engaged in rubbing one another. In both the baths, the greater number of the men and the women were stripped of all clothes; but a few wore a small handkerchief round their waists. The whole spectacle reminded me strongly of those fiery pictures of "le anime nel purgatorio," which one meets at the door of almost every church in Italy, over the begging or alms-box." 199.

### Leibenstein.

This is rather a celebrated spa, situated near Meiningen, the birth-place of our Dowager Queen. The waters contain a considerable portion of iron, being about a seventh part of the whole solid contents—fourteen grains in the pint. The other matters are slightly aperient. "It is heating to excess, over-exciting and bending, and likely to produce eruptions." Dr. G. thinks "it resembles in its effects that heterogeneous compound, commonly called Griffith's mixture." If this be the fact, we have little hesitation in saying that Leibenstein is one of the most valuable spas in Germany. It is mostly used as a bath, the temperature being about 50°, raised up by additions to a proper heat.

### Kissengen.

This spa is rising fast in reputation. It is only a day's journey from Liebenstein, and seventeen German miles from Frankfort. Dr. Maas is the medical superintendant. The most famous spring is called the Ragozi, lately discovered.

"The *Ragozi* enjoys already an European reputation. It emerges from a depth of twelve feet, through rounded pebbles and basaltic stones, at a temperature one degree higher than that of the *Maxbrunnen*. Its taste is sharp, strongly saline, and acidulous, with a smack also of a saline, bitter, and rough vitriolic nature. It reminded me of the stronger chalybeate water near Rosenstein, at Stuttgardt. Nor is it surprising that it should so taste, seeing that it contains nearly three-quarters of a grain of carbonate of iron in sixteen ounces. When the *Ragozi* is found to affect the head, the glass filled with it, is previously immersed into hot water, so as to allow the greater part of the gas to escape. If, after this, the *Ragozi* should still be found to affect the head, then the *Pandur* is to be substituted for it. But to the prolonged use of the latter spring Dr. Maas objects that it weakens the stomach." 289.

The solid contents of a pint of this water are ninety grains. It is considered a panacea for chronic diseases. It is "gently purgative, depurative, and alterative."

"Judging, *primâ facie*, from such a circumstance alone, I should expect the very best effects from the use of the *Ragozi* in every possible modification of complaints of the stomach—from the mere want of appetite and oppression after eating, to the most complicated derangement of the various abdominal processes which constitute digestion. I will only instance, as an example, the nausea and retching (first indications of forthcoming disease) which salute the overnight-tippler, when he first wakes in the morning. Half a glass of the *Ragozi*, taken immediately on waking, will arrest those symptoms." 291.

The *Ragozi* removes impurities from the intestinal tube, while it gives tone to the stomach and bowels. But it is in sterility that Kissengen has acquired great reputation. There is another class of invalids who are said to derive much benefit from Kissengen, " hard livers"—such as have a hard liver from having lived hard." Some remarkable instances were related to Dr. Granville by Dr. Maas, where livers as hard as stone had been softened down by the Kissengen waters. These hard-liver stories, however, are to be swallowed, *cum grano salis.*

We now proceed to the spas of Nassau—nor shall we dwell long on waters which have become so well known through the medium of the "*old man of the Brunnens.*" FACKINGEN and GEILNAU, on opposite banks of the river Lahn, were the first two places visited by our author. The water of Fackingen is supposed to be the strongest alkaline water in Germany. But it is little frequented. Geilnau is of lower temperature than the Seltzer waters, but is nearly the same in composition, as to solid contents, and contains much more carbonic acid. Great quantities of these waters are now exported to other countries.

### WEISBADEN.

This is the capital of Nassau, distant only two stages from Frankfort, and delightfully situated in a beautiful valley of the Tanus mountains. The Weisbaden waters have been justly compared to weak chicken broth slightly salted—but not quite so nutritious. They emit an odour not unlike that of lime while in the act of being slacked. Here, as at Baden Baden, the mania of gambling rages in a fierce degree!

" One of the principal effects to be noticed in using the mineral water at Wiesbaden, is the great change that supervenes in the state of the skin. From being dry and ruvid, it becomes moist and soft. Sometimes only a little redness of the skin, at other times a slight eruption, ensues, in the course of bathing; and through this series of simple phenomena alone, many chronic disorders have been cured.

If a patient bathes for old gouty and rheumatic pains, he will often find them increased first, and become more intense; his walk will be more difficult, the joints more stiff, the muscles exceedingly sore. In eight or ten days these symptoms will disappear, and recovery will follow after the full course of the waters.

The appetite and functions of the stomach are generally augmented after a few baths. The action of the intestines is sufficiently promoted by the proper and internal use of the water. The Wiesbaden water is held, with some justice, to be of a purgative quality. When the baths have not been injudiciously recommended, sleep is generally improved by them, it becomes uniform and regular, and is not disturbed by dreams. The result of such a regularity is an augmentation of vital energy in the patient. It is, however, of the utmost importance, not to allow sleep to overcome the patient while in the bath. When it does so, further bathing would be a source of danger, without properly consulting a physician upon that point. Such a sleep, to say the least, is suspicious, and is generally accompanied by a heavy and full pulse, as well as by a tendency to giddiness.

In cases of paralysis I found the baths of Wiesbaden of an efficacy nearly equal to that possessed by Toeplitz. A very remarkable case of recovery from a total suspension of the faculty of moving the limbs, unconnected with any rheumatic affection, came under my notice three years ago; Dr. Peez related to me several other diseases of a similar kind.

The internal use of the *Kochbrunnen,* or of the *Schützenhof,* will be found very successful—much more so than Cheltenham or Leamington—in all cases of congestion, fulness, and obstruction in any of the abdominal viscera of both sexes. The baths must in all these cases be combined with the internal use of the water, and if the subject be a female, the necessity for bathing is still more urgent." 417.

The waters of Wiesbaden are supposed to be almost specific in hypochondriacism—a picture of which is presented by Dr. Granville. The water is drank early in the morning, on an empty stomach, in the quantity of four to six tumblers. Breakfast follows half an hour after drinking the waters, and bathing half an hour after that meal. This is a practice which cannot be good, if the waters united all the good qualities of all the mineral springs in the earth. The breakfast here recommended can hardly deserve the name—"a cup of coffee or chocolate, with milk, or a basin of soup, with the yolk of an egg, will be sufficient." This is a "baberwash" breakfast indeed!

From Wiesbaden, our author proceeded to the far-famed

### SCHLANGENBAD,

Or serpent bath, immortalized by the "Old Man of the Brunnens."

"The Schlangenbad water is said to soften the skin. It does so only like any other water in which the larger proportion of its saline ingredients consists of carbonate of soda and magnesia. Such is the case with the Schlangenbad. The notion that this peculiar quality of the water is due to the animal matter derived from the snakes, or say rather little vipers (for they are nothing else), is absurd; for after the most scrupulous analysis, no traces of animal matter can be detected in the water." 446.

"This water contains in every pint three grains of carbonate of soda. If we suppose, therefore, a bath to consist of seventy gallons, or 560 pints of the water (taking it for granted that all the water supplied is genuine), it will hold, in solution, exactly three ounces and a half of carbonate of soda; besides which it will contain nearly an ounce of carbonate of magnesia. Now let my fair readers (if I should be fortunate enough to have any) cause an equal amount of those two saline combinations to be dissolved in their tepid baths, and I will answer for the consequences, as far as the lubrification and satinization of their skin are concerned. But with regard to the promised delight from the baths at Schlangenbad, neither their natural temperature, nor their composition, could be expected to induce any such feeling. The sensations experienced in them appear to me to amount to no more than the natural sense of comfort, generally felt in summer, upon entering a tepid bath. In conclusion, when I set myself to compare Schlangenbad with other thermal springs, and especially with Wildbad, I frankly confess that I felt great disappointment at the result of my enquiries." 448.

The medicinal virtues of the Serpent water, when swallowed, are so slight that we need not even mention them. Thus vanishes the cosmetic reputation of the Schlangenbad! The "Bubbles," however, of Nassau, have proved a *sovereign* beautifier of the sexagenarian author, and, like a dip in Medea's cauldron, have made a *young* man of him—a knight—Poor-law Commissioner—Lieutenant Governor—Baronet!

SCHWALBACH.

There are three springs here. The water in all of them is clear as crystal, slightly bubbling, and of pleasant acidulous taste. They are all chalybeate, and unite with the taste of iron that of acidulated Seltzer water. It lies heavy and cold on the stomach, especially the Wemenbrunnen. We need not dwell on the wondrous cures performed by these waters.

"My experience distinctly authorizes me at the same time to declare, that the worthy *inspecteur des eaux* has carried the list of ailments which the Schwalbach waters are said to cure, beyond even the limits of credulity. That list reminds one of the printed envelopes put around that celebrated cosmetic which owes its birth to the filthiest and most offensively smelling city in Europe." 70.

By this we suppose Dr. G. means Cologne, which is certainly a dirty town, though by no means so malodorous as some cities of classic Italy. Sir F. Head, however, has driven the French and German families "slick right away" from Schwalbach, and colonized the Spa with some 3000 British visitors annually!

EMS.

This Spa, Dr. G. thinks, is in a false position, not only as a place of residence in the Summer, but as regards the "true point at which the mineral sources ought to have been converted to use." This Spa, so much frequented by the English, of late, is not held in much estimation by our author.

"In fact, I may state it at once and openly, that Ems is half a century behind every one of the Spas of the first class that I have seen in Germany and described in the present volumes." 502.

The principal drinking springs are the Kraenchenbrunnen, and Kesselbrunnen. The temperature is about 24° of Reaumur.

"The water is clear, and has no smell. A considerable quantity of gas escapes on taking up the water with a glass. According to Struve, a pint of sixteen ounces contains fifteen and a half cubic inches of free carbonic acid. The taste, which is gently sub-acid, and milk-warm at first, is succeeded in the mouth by one of an alkaline and soapy nature, which arises from a large proportion of carbonate of soda being left behind, quite insulated, by the evaporation of the gas. The place in which the water is drunk is not calculated to inspire the patients with any cheering thought of future recovery. It is, moreover, so confined that no more than three or four persons can approach it at the same time conveniently." 503.

The bath, Dr. G. avers, occasions at first, a sense of lassitude and drowsiness—next, relaxation of the pores—and lastly a red efflorescence on the skin, called the *bath-itch*. The waters have very little aperient effect, and English patients generally fly to purgatives, in conjunction with the waters.

"This habit of taking aperient medicines so prevalent among English people, astonishes all foreigners on the continent, who are still more astonished when they visit England, and see, or are told of, the basketsful of drugs which enter, almost daily, a gentleman's house in London. One of these foreign visiters asked me one day the reason of such a practice, and of the difference which existed in that respect between the English and continental nations. In answer

I pointed, as we were walking along, to the juicy barons of beef on the butcher's shambles; to the white square loaves of the bakers, with alum and lime in them; to the scantiness and high prices of the *legumes* in the markets; to the fiery port and sherry proclaimed at the door of wine vaults; to the XXX and Burton ales; to the Whitbread entire and Guinness stout; in fine, to the smoke and atmosphere of London: adding, that the fashion of pills and draughts' taking in England, after all, was of the upper and better classes of society only; and by no means to be observed among the multitude." 509.

Dr. Kreysig avers that these waters have been very useful in pulmonic complaints—nervous debility—female complaints—indigestion. Our author, as was said before, has no high opinion of these waters, though he has often prescribed them. He considers them as alteratives rather than aperients—as "disturbing waters"—which rarely cure diseases till some other Spa has been resorted to. We have lying before us a little work on the Waters of Ems by Dr. Thilenius, in which a very different account of this Spa is given. Taking the Doctor's statements "*cum grano salis*," we would still be inclined to attach some importance to these waters.

The disturbance of the nervous system, caused by these waters, is attributed by Dr. Granville to the large quantities of soda which they contain. As much as a drachm of this alkali is taken in the usual morning dose of the Ems Spa. The air of the narrow valley, too, in which the Spa is situated, cannot be very conducive to health.

"Hence Ems can never suit an hypochondriac, no matter from what functional disorder his unhappy state may arise. It never can suit persons labouring under any modification whatever of disease of the heart, whether structural or merely functional. It never can suit such females as have been suffering from repeated losses, have become blanched, weak, and shaky, and whose head feels heavy yet light, confused yet empty, always on the *qui vive* yet oppressed;—an anomalous condition, inexplicable on any known physiological principle, yet nevertheless too true—as those who have exercised for twenty years the particular branch of the profession I exercised in London, until within the last four years, can readily testify." 512.

This last expression has rather surprised us, as we thought Dr. G. was as deeply immersed in obstetric practice as he ever was.

We must now bring our article to a close, having, as carefully as possible, gleaned from these volumes the chief professional information which they contain. Nine tenths of Dr. Granville's pages are occupied with anecdotes, personal narratives, descriptions of scenes, localities, men, manners. &c. Into these we dared not enter, though they will be greedily perused by general readers. We would advise Dr. Granville, in a new edition, to leave out a great deal of merely temporary matters, and to condense the two volumes into one, so as to make the work a stock-book, or, as we said before, a STARK-BOOK for Spa-tourists in Germany. The extracts which we have taken from the work, will shew that Dr. Granville writes in a light, pleasing, often humorous style—not always free, however, from a little spice of self-importance, which some ill-natured critics might be tempted to characterize as vanity. This is so venial a fault, that it is scarcely worth notice. If he will take the hint we have thrown out, respecting condensation in a second edition, we venture to aver that he will NOT have cause to complain—

——————— Dum brevis esse laboro

Obscurus fio.

